# Acute muscle damage responses in elbow flexors following eccentric quasi-isometric exercise

**DOI:** 10.1038/s41598-026-43855-4

**Published:** 2026-03-13

**Authors:** Yu-Chin Lin, Tsung-Lin Chiang, Shih-Hsuan Chan, Chih-Hsiang Hsu, Huey-June Wu

**Affiliations:** 1 Graduate Institute of Sport Coaching Science, Chinese Culture University, No. 55, Hwa- Kang Rd., Yang-Ming-Shan, Taipei City, 11114 Taiwan (R.O.C.); 2 Physical Education Office, National Taipei University of Technology, No.1, Sec. 3, Zhongxiao E. Rd., Da-an District, Taipei City, 106 Taiwan (R.O.C.)

**Keywords:** Resistance exercise, Exhaustive exercise, Muscle fatigue, Mechanical tension, Contraction characteristics, Health care, Medical research, Physiology

## Abstract

This study aimed to compare acute muscle damage responses of the elbow flexors following eccentric quasi-isometric (EQI) and eccentric (ECC) exercise. Thirty healthy young men were randomly assigned to EQI or ECC (*n* = 15/group). Participants performed five sets of dumbbell elbow flexion to volitional failure. Maximal voluntary isometric contraction (MVC), elbow joint range of motion (ROM), upper arm circumference (CIR), pressure pain threshold (PPT), plasma creatine kinase (CK), and myoglobin (Mb) were measured before, immediately after, and 1, 2, 3, and 7 days post-exercise. Time under tension (TUT) was recorded. TUT was approximately 3.5-fold longer in EQI than ECC. ECC induced greater reductions in MVC (− 30% vs. −15%) and ROM (− 27% vs. −13%), and larger increases in CIR (+ 5% vs. +2%). Peak CK (11,601 ± 9,483 vs. 135.2 ± 33.1 IU/L) and Mb (406.7 ± 271.8 vs. 17.7 ± 6.2 ng/mL) were markedly higher following ECC. Distal PPT was 13–19% lower in ECC. Most variables returned to baseline by day 7 in EQI. Despite longer TUT, EQI induced substantially less acute muscle damage and faster recovery than ECC when both were performed to volitional failure at the same relative external load.

## Introduction

Eccentric (ECC) exercise is widely utilized to enhance neuromuscular function and physical performance. However, its application is often limited by a high risk of muscle damage and prolonged recovery, which may be unfavorable for many populations^1–3^. The likelihood of exercise-induced muscle damage (EIMD) is particularly elevated when ECC exercise is performed at high intensity^4^, high velocity^5^, or at extended muscle lengths^6,7^. EIMD is characterized by a constellation of symptoms, including acute strength loss, delayed onset muscle soreness (DOMS), muscle swelling, restricted range of motion (ROM), increased perceived soreness, altered pressure pain threshold (PPT), and elevated levels of circulating muscle damage markers such as creatine kinase (CK) and myoglobin (Mb)^1,8,9^. The duration and severity of recovery following EIMD are primarily determined by the initial extent of muscle damage, which is modulated by both the exercise dosage and muscle length during contraction^10^. In untrained individuals, excessive eccentric loading may even induce severe myofibrillar disruption or lead to rhabdomyolysis^11^.

Recently, a novel contraction mode termed eccentric quasi-isometric (EQI) has been proposed, integrating isometric (ISO) and ECC characteristics. In this paradigm, a sustained submaximal isometric contraction is first performed at a fixed joint angle until task failure, followed immediately by an extremely slow eccentric lengthening (~ 1.3°·s⁻¹) while the individual continues to voluntarily resist the load, attempting to maintain the ISO posture throughout the range of motion^12–15^. This technique has been shown to elicit a prolonged time under tension (TUT) in the targeted muscle without the need for high repetition volumes^14,15^. The gradual force decline under sustained loading in EQI allows for the application of high mechanical tension while avoiding sudden force spikes, thereby potentially reducing stress on tendons and joint structures^16^. It is hypothesized that this unique contraction profile facilitates concurrent accumulation of mechanical and metabolic stimuli within a single repetition, possibly enhancing hypertrophic, neuromuscular, and tendinous adaptations through mechanotransduction mechanisms^12,14,17,18^. Given its ability to provide high loading stimuli without requiring ballistic movement or high training volumes, EQI may offer a viable training option in rehabilitation contexts^19,20^ or for populations unable to tolerate traditional high-intensity ECC training. Despite these theoretical advantages, empirical evidence on EQI remains scarce, particularly concerning its potential to induce acute muscle damage and the underlying physiological mechanisms.

From a muscle damage perspective, the severity of exercise-induced muscle damage is strongly influenced by eccentric contraction velocity, muscle length, and the rate of force application. Faster eccentric actions and lengthening at longer muscle lengths have consistently been shown to induce greater myofibrillar disruption, strength loss, and elevations in circulating muscle damage markers^4,6^. In contrast, slow-velocity, submaximal eccentric contractions are associated with greater metabolic stress but a lower propensity for structural muscle damage^5^. Given that EQI is characterized by a prolonged isometric phase followed by an extremely slow eccentric lengthening without abrupt force spikes, it is theoretically plausible that EQI would impose a lower acute muscle damage stimulus than traditional eccentric exercise, despite a longer time under tension^12,14^.

While most empirical evidence on eccentric quasi-isometric (EQI) exercise has centered on lower limb extensor groups—particularly the knee extensors^13–15^ and plantar flexors^21^—its applicability to fusiform upper limb muscles, such as the biceps brachii, remains poorly understood^12,22^. Due to differences in fiber arrangement and habitual loading patterns, fusiform muscles may respond differently to EQI-induced tension compared to pennate lower limb muscles^23^. Indeed, previous studies have reported that elbow flexors are more susceptible to ECC induced muscle damage than pennate muscles such as the knee extensors^1^. Despite the proposed mechanical advantages of EQI in prolonging time under tension and avoiding abrupt load spikes, it remains unclear how variations in tension application rate or angle-specific loading phases influence microdamage in fusiform muscles during EQI. Evidence suggests that eccentric contractions performed at longer muscle lengths induce greater structural damage to muscle fibers^24^, and recent findings indicate that EQI protocols may result in distinctive patterns of damage distribution in the knee extensors^14^. However, whether such patterns extend to the elbow flexors under EQI loading conditions is yet to be empirically verified.

Moreover, recent evidence suggests that functional and biochemical markers of muscle damage exhibit distinct recovery trajectories and are not always closely aligned. Deyhle et al.^25^ reported that the overlap in gene expression patterns associated with peak soreness, strength loss, and serum CK elevation was less than 1%, indicating a poor molecular convergence among these commonly used indices. Similarly, it has been demonstrated that these markers are not linearly correlated, and reliance on a single marker may not accurately reflect the overall magnitude or progression of exercise-induced muscle damage^26^. Therefore, a multimodal assessment combining functional and biochemical indicators is necessary to accurately characterize the acute physiological response to EQI exercise.

In summary, this study aimed to compare the muscle damage responses of the elbow flexors following EQI and ECC contractions. It was hypothesized that both contraction modes would induce typical eccentric damage responses, peaking at 24–72 h and resolving within 7 days. Compared to ECC, EQI was expected to elicit smaller impairments in muscle function and lower elevations in biochemical markers. The findings may help evaluate the short-term safety and underlying mechanisms of EQI, providing practical insights for optimizing resistance training prescriptions.

## Materials and methods

### Participants and study design

Thirty healthy young men participated in this study. None had engaged in regular resistance, aerobic, or flexibility training in the past year. To minimize inter-individual variability in baseline measures and exercise-induced responses, only male participants were included, as previous studies have reported sex-related differences in the extent of eccentric exercise-induced muscle damage^22,27,28^. Participants were free from any history of upper limb musculoskeletal, joint, or bone injuries. Their mean ± SD age, height, body mass, and one-repetition maximum (1RM) of the target exercise were 20.3 ± 0.7 years, 174.1 ± 6.4 cm, 68.9 ± 10.8 kg, and 15.4 ± 4.0 kg, respectively. This study was conducted in accordance with the Declaration of Helsinki and relevant guidelines and regulations. The protocol was approved by the Institutional Review Board of Fu Jen Catholic University (FJU-IRB No: C108083), the contracted IRB for Chinese Culture University. All participants provided written informed consent prior to participation. The study was registered at ClinicalTrials.gov (Date: 25/2/2026, ID NCT07431879; https://clinicaltrials.gov/study/NCT07431879).

Participants (*n* = 30) were randomly assigned based on their 1RM values to perform either EQI or ECC (*n* = 15 per group). Each participant was further randomized to perform all testing using either their dominant or non-dominant upper limb. Muscle damage markers were assessed at six time points: baseline (Pre), immediately post-exercise (P0), and at 1 (D1), 2 (D2), 3 (D3), and 7 (D7) days post-exercise. The dependent measures included MVC, ROM, relaxed arm circumference (CIR), PPT, serum CK, and Mb levels. All experimental procedures were conducted in a temperature-controlled laboratory (24–26 °C) at the same time of day for each participant to avoid circadian rhythm effects. Participants were instructed to refrain from strenuous physical activity, unfamiliar exercise, and the use of anti-inflammatory medications or supplements (e.g., NSAIDs, protein, amino acids) throughout the study period. They were also advised to avoid alcohol, caffeine, and analgesics, and to abstain from any interventions targeting the exercised muscles (e.g., massage, stretching, thermal or cryotherapy). Adequate hydration was emphasized following the intervention to minimize potential risks of rhabdomyolysis-related renal complications. A priori power analysis was conducted using G*Power software (version 3.1.9.7), based on pilot data comparing D1 MVC between ECC and EQI groups (Cohen’s *d* = 1.31). With an estimated effect size of *f* = 0.40, alpha level set at 0.05, and power (1-*β*) of 0.80, the minimum required sample size per group was determined to be 10. To account for potential attrition during the study period, 15 participants were recruited per group.

### Familiarization sessions

Two familiarization sessions were scheduled prior to the formal experiment. The first session was conducted one week before the experimental trial and included an overview of the study procedures, instruction on the EQI and ECC movement techniques, and explanations of the assessment protocols. The second session took place three days before the main trial and focused on detailed instruction and practical rehearsal of the EQI and ECC exercises, as well as the measurement protocols for MVC, ROM, CIR, and PPT. All procedures were verbally explained and demonstrated by the research staff. Participants practiced the experimental protocol under unloaded conditions to simulate the testing environment. This process was designed to minimize the potential influence of learning effects on the experimental outcomes.

### One-repetition maximum testing

The 1RM of the elbow flexors was determined using a unilateral dumbbell biceps curl and served as the basis for subsequent exercise intensity prescription. During the test, participants were seated on a preacher curl bench specifically designed for the biceps. The seat was adjusted to ensure that the axilla was firmly supported by the pad, with the upper arm angled approximately 45° relative to the trunk and the elbow flexed at 90° as the starting position. To promote neuromuscular activation and reduce the risk of injury, a two-stage warm-up protocol was implemented. In the first stage, participants performed 5–10 repetitions using a self-selected load corresponding to 40%–60% of their perceived 1RM. After a brief rest, the second stage involved 3–5 repetitions using 60%–80% of the perceived 1RM. For the 1RM determination, participants attempted a single repetition with a load they subjectively estimated as their maximal effort. A successful attempt was defined as completing a full elbow flexion movement from the starting angle without compensation or assistance. If the attempt was successful, the load was increased and re-tested after a short rest; if unsuccessful, the load was reduced. Each participant was required to complete a valid 1RM within five attempts. If unsuccessful after five trials, the test was terminated and rescheduled. The final successful load was recorded as the 1RM and used to determine the subsequent training intensity.

### EQI and ECC exercises

Participants performed the exercises using either their dominant or non-dominant arm, as determined by random assignment. All exercises were conducted in a seated position on a preacher curl bench with the shoulder flexed to approximately 45° and no abduction (0°). The dumbbell load was standardized at 70% of each participant’s 1RM. Prior to each repetition, the researcher assisted in positioning the dumbbell at the starting position with the elbow flexed at 90°, allowing the participant to perform the EQI or ECC movement independently. Verbal encouragement was provided using standardized cues to reduce premature termination due to motivational factors.

#### Eccentric quasi-isometric exercise

Participants began each repetition at 90° elbow flexion and were instructed to exert maximal bracing effort to maintain the joint angle against the external load while avoiding any force that could initiate a concentric contraction. As fatigue accumulated, the inability to maintain the joint position resulted in an eccentric displacement, during which participants continued to resist the load as forcefully as possible while repeatedly attempting to re-establish the isometric hold until the elbow reached full extension (0°). A single repetition was defined as one continuous task from the onset of the initial isometric contraction at 90° to full elbow extension, characterized by a transition from attempted isometric holding to eccentric lengthening due to fatigue, constituting one full EQI contraction^12,14^. TUT was recorded using a handheld digital stopwatch (Seiko, Tokyo, Japan) from the initiation of the isometric hold at 90° until the dumbbell reached full elbow extension (0°). Participants completed 5 sets of 1 repetition, with 90 s of rest between sets.

#### Eccentric exercise

Participants began at the same 90° elbow flexion starting position and performed repeated eccentric lowering movements until failure, defined as the inability to complete the movement through the full ROM or maintain the prescribed tempo. All repetitions were performed at a controlled cadence of 2.5 s per eccentric phase, guided by a metronome. Each set was terminated once the participant could no longer resist the load or keep pace with the metronome. A total of 5 sets were completed, with 90 s of rest between sets. TUT was calculated by multiplying the total number of completed repetitions in a set by 2.5 s.

### Maximal voluntary isometric contraction torque

MVC of the elbow flexors was assessed using an isokinetic dynamometer (System 4 Quick-Set™, Biodex Medical Systems, Shirley, NY, USA). Participants were seated and securely stabilized using a pelvic strap and two shoulder harnesses to minimize contribution from other body segments. The shoulder was positioned at 45° of flexion and 0° of abduction, with the forearm supinated. Participants held the handle attached to the dynamometer lever arm, which was aligned with the elbow joint axis. The elbow was fully extended at 0°, and testing was conducted at 90° of elbow flexion. Participants were verbally encouraged to exert maximal effort during each 3-second isometric elbow flexion. Three trials were performed, each separated by 45 s of rest. The peak torque value during the 3-second contraction period was recorded for each trial, and the highest of the three values was used for further analysis^29^.

### ROM of the elbow joint

Elbow joint ROM was assessed using an 8-inch manual goniometer (EMI Plastic Goniometer, Elite Medical Instruments, Fullerton, CA, USA). Three anatomical landmarks were marked with an oil-based pen: the lateral midline of the humerus, the lateral epicondyle of the elbow (axis), and the midline between the ulna and radius. Landmarks were verified before each measurement to minimize placement error. Participants stood upright with feet shoulder-width apart, arms fully relaxed, and eyes facing forward. Two joint angles were recorded: the relaxed elbow joint angle (RANG) with the arm hanging naturally, and the fully flexed elbow joint angle (FANG) during active flexion. Each position was measured at least three times with 15 s of rest between trials. The mean of the three values was used for analysis. If any value differed by more than 2 cm, an additional measurement was taken. ROM was calculated by subtracting FANG from RANG^29^.

### Upper arm circumference

CIR was measured using a flexible measuring tape while participants stood in a relaxed posture with their arms fully extended and hanging naturally at their sides. The measurement site was defined as the midpoint between the acromion process and the lateral epicondyle of the humerus. All measurements were performed three times by the same investigator, and the average of the three values was used for statistical analysis^29^.

### Pressure pain threshold

PPT was assessed using a digital pressure algometer (Wagner Force Ten™ FDX 50, Wagner Instruments, Greenwich, CT, USA) at the distal (3 cm; PPT_dist_), middle (9 cm; PPT_mid_), and proximal (15 cm; PPT_prox_) sites above the antecubital crease along the biceps brachii. Participants lay in a supine position with the arm relaxed and the forearm supinated. A 1.0 cm² probe was applied perpendicularly to the skin surface. Pressure was manually increased at a target rate of approximately 50 kPa·s⁻¹, which was visually monitored in real time using the algometer digital display. Prior to data collection, the assessor underwent standardized training and repeated practice to ensure a consistent and reproducible loading rate close to the target value. Participants were instructed to verbally indicate (“stop”) the first sensation of pain, at which point the applied pressure was immediately released. The corresponding pressure value (kPa) was automatically recorded by the device. Measurements were performed in a standardized order from distal to proximal sites (3 to 15 cm) with 10-second intervals between trials. Two measurement rounds were conducted with a 5-minute rest interval. The average of three trials per site was used for subsequent analyses^30^.

### Plasma CK activity and myoglobin concentrations

Approximately 5 mL of venous blood was withdrawn using a standard venipuncture technique from the cubital fossa region of the non-exercised arm. Blood samples were centrifuged for 10 min to separate plasma, and the plasma samples were stored at −80 °C until analyses. Plasma CK activity was analyzed spectrophotometrically using an automated clinical chemistry analyzer (AU5820, Beckman Coulter Inc., USA). Plasma myoglobin concentration was measured using a chemiluminescent immunoassay on an automated immunoassay analyzer (DXI800, Beckman Coulter Inc., USA).

### Statistical analyses

Independent-samples t-tests were conducted to compare baseline values of all variables between the ECC and EQI groups. Data normality for each variable was assessed using the Shapiro–Wilk test, and homogeneity of variances was examined using Levene’s test. A two-way mixed analysis of variance (ANOVA) was conducted to evaluate the effects of group (ECC and EQI) and time (Pre, P0, D1, D2, D3, and D7) on each dependent variable. When significant interactions were found, Tukey’s post-hoc analyses were performed. Effect sizes were determined by partial eta squared (partial *η*²), interpreted as small (~ 0.02), moderate (~ 0.13), or large (> 0.26) effects (Bakeman, 2005). Between-group effect sizes were calculated using Cohen’s d, with values interpreted as small (*d* < 0.5), medium (*d* = 0.5–0.8), or large (*d* > 0.8) (Cohen, 2013). The level of statistical significance was set at *p* ≤.05. The data are presented as mean ± standard deviation (SD).

## Results

No significant differences (*p* >.05) in any of the baseline dependent variables were found between the ECC and EQI groups (Table 1).

**Table 1:** Baseline values (mean ± SD) of maximal voluntary isometric contraction (MVC) torque, range of motion (ROM), upper arm circumference (CIR), pressure pain threshold (PPT), plasma creatine kinase (CK), and myoglobin (Mb) at baseline for the ECC and EQI groups.

Variables	ECC	EQI
1RM (kg)	15.9 ± 3.7	15.4 ± 4.0
MVC (Nm)	54.81 ± 13.49	58.90 ± 9.09
CIR (cm)	26.76 ± 1.06	26.64 ± 2.23
PPT_dist_ (kPa)	70.33 ± 23.09	67.50 ± 20.58
PPT_mid_ (kPa)	67.00 ± 22.16	61.36 ± 24.19
PPT_prox_ (kPa)	61.93 ± 22.55	57.14 ± 22.81
ROM (°)	119.44 ± 5.85	120.67 ± 6.76
CK (IU/L)	91.43 ± 36.77	110.46 ± 31.48
Mb (ng/mL)	14.01 ± 4.54	14.72 ± 3.99

### Time under tension in EQI and ECC

**Fig. 1:**
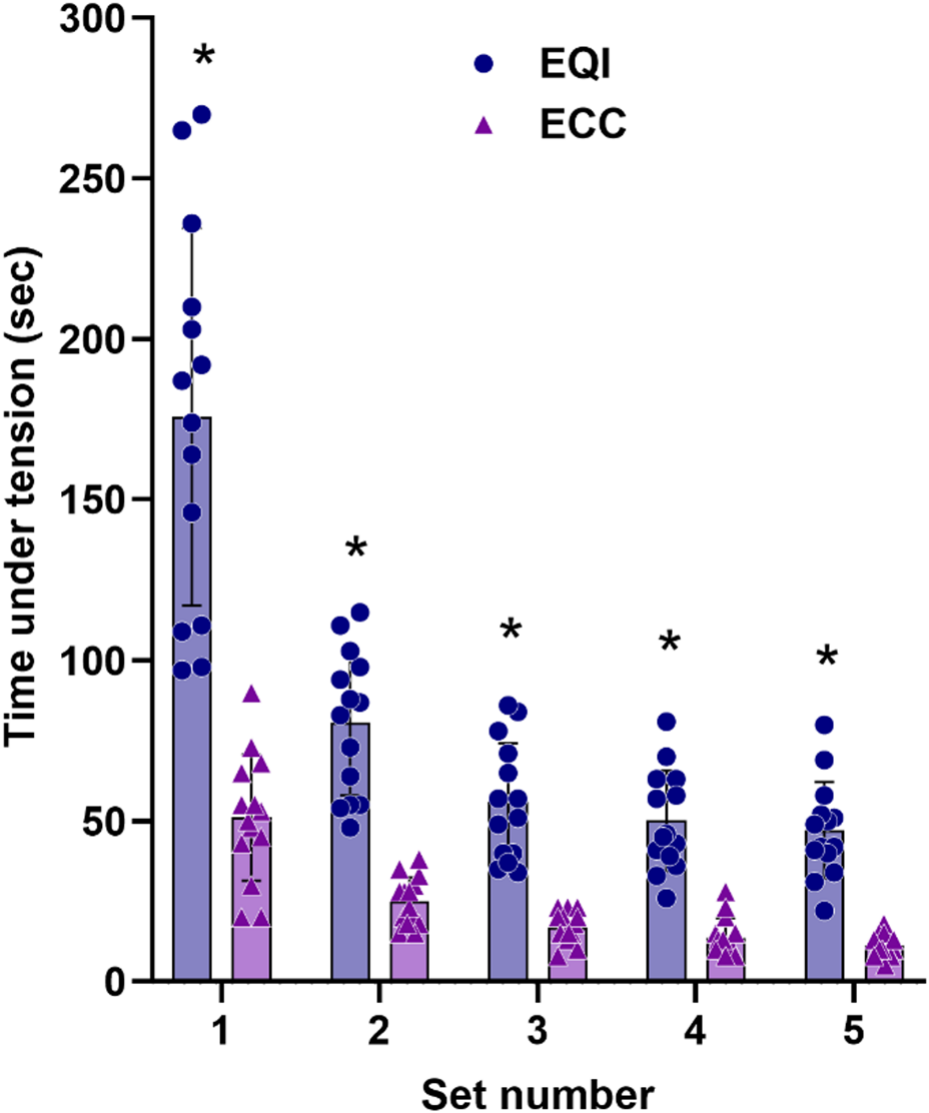
Time under tension (TUT; mean ± SD) across the five exercise sets in the eccentric quasi-isometric (EQI) and eccentric (ECC) groups. * indicate significant differences between groups at the corresponding set (*p* <.05).

TUT was significantly greater in the EQI compared to the ECC across all five sets (*p* <.001). The TUT values for sets 1 to 5 were as follows: set 1 (185.33 ± 71.54 s vs. 59.40 ± 12.75 s), set 2 (81.47 ± 23.63 s vs. 26.47 ± 8.59 s), set 3 (56.47 ± 18.22 s vs. 17.40 ± 5.49 s), set 4 (52.87 ± 19.27 s vs. 15.93 ± 7.74 s), and set 5 (48.00 ± 16.40 s vs. 11.20 ± 3.80 s), for EQI and ECC groups respectively (Fig. 1).

### Maximal voluntary isometric contraction (MVC) torque

**Fig. 2:**
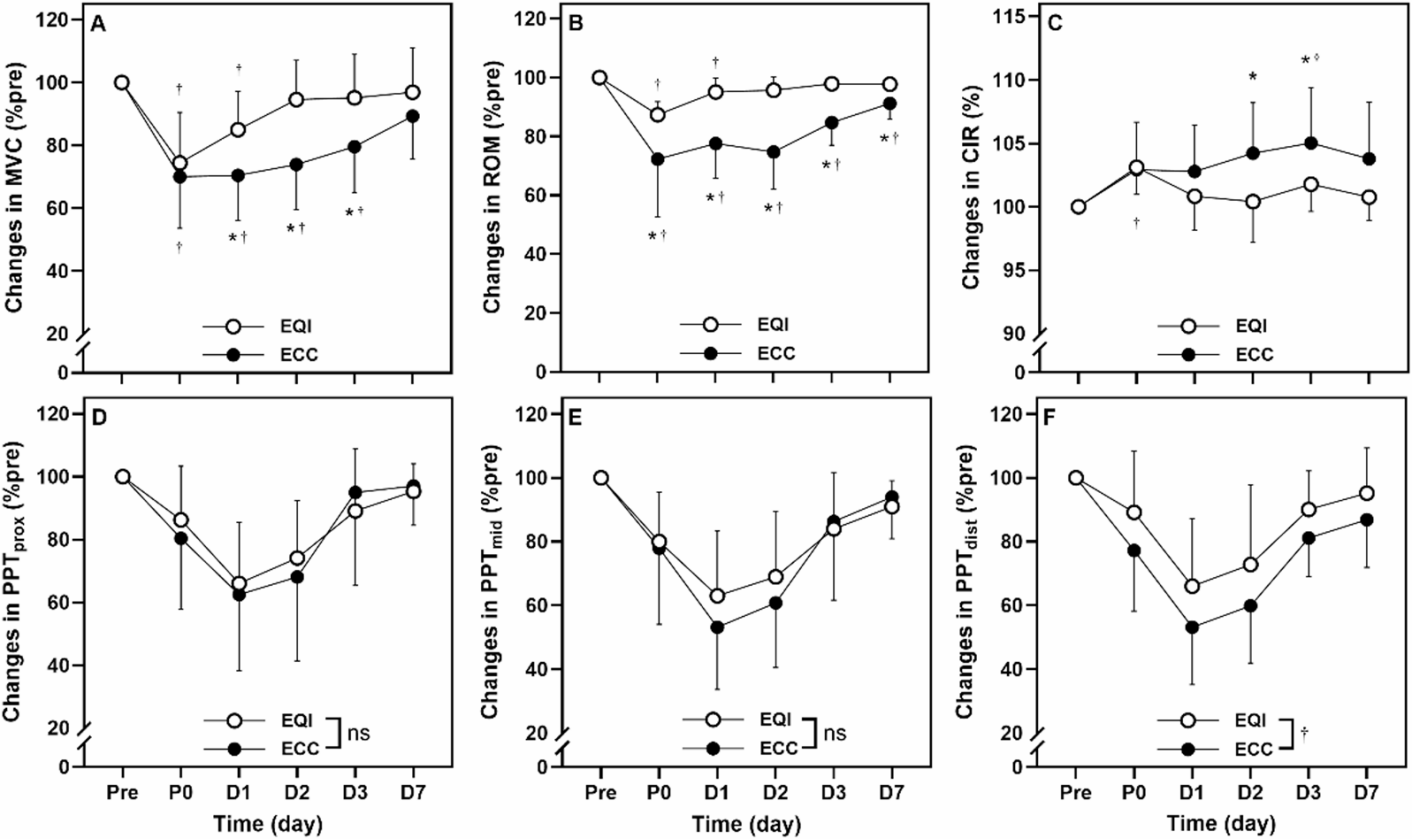
Normalized changes (mean ± SD) from baseline (Pre = 100%) in **A** maximal voluntary isometric contraction (MVC), **B** range of motion (ROM), **C** circumference (CIR), pressure pain threshold at the **D** proximal (PPT_prox_), **E** middle (PPT_mid_), and **F** distal (PPT_dist_) sites of the elbow flexors measured before (Pre), immediately after (P0), and 1 (D1), 2 (D2), 3 (D3), and 7 (D7) days following the eccentric (ECC) and eccentric quasi-isometric (EQI) exercise interventions. * indicate significant differences between groups (*p* <.05); ^†^ indicate significant differences compared to Pre within the same condition (*p* <.05); “ns” indicates no significant difference.

A significant group× time interaction was observed for MVC (*p* =.007, *η*² = 0.109). MVC was significantly lower in the ECC than in EQI at D1 (*p* =.007, *d* = −1.090), D2 (*p* <.001, *d* = −1.528), and D3 (*p* =.007, *d* = −1.090). In the ECC group, MVC decreased significantly from Pre at P0, D1, D2, and D3 (*p* <.001, *η*² = 0.545). In contrast, reductions in EQI were observed only at P0 and D1 (*p* <.001, *η*² = 0.421) (Fig. 2A).

### Range of motion (ROM) of the elbow joint

A significant group× time interaction was observed for ROM (*p* <.001, *η*² = 0.203). ROM was significantly lower in the ECC group than in EQI at P0 (*p* =.020, *d* = −0.921), D1 (*p* <.001, *d* = −1.690), D2 (*p* <.001, *d* = −1.932), D3 (*p* <.001, *d* = −1.831), and D7 (*p* =.003, *d* = −1.222). In ECC, ROM significantly decreased from Pre at all time points from P0 to D7 (*p* <.001, *η*² = 0.551). In EQI, reductions were observed only at P0 and D1 (*p* <.001, *η*² = 0.683) (Fig. 2B).

### Upper arm circumference (CIR)

A significant group× time interaction was observed for CIR (*p* =.003, *η*² = 0.151). CIR was significantly greater in the ECC group than in EQI at D2 (*p* =.033, *d* = 0.930) and D3 (*p* =.026, *d* = 0.971). In ECC, CIR was significantly higher at D3 compared to Pre (*p* =.002, *η*² = 0.290). In EQI, CIR was significantly lower at P0 than Pre (*p* <.001, *η*² = 0.345) (Fig. 2C).

### Pressure pain threshold (PPT)

No significant group× time interaction was found for PPT_prox_ (*p* =.699, *η*² = 0.022). A significant main effect of time indicated that values at P0, D1, and D2 were lower than Pre (*p* <.001, *η*² = 0.420) (Fig. 2D).

For PPT_mid_, no significant interaction was observed (*p* =.336, *η*² = 0.041). A time main effect showed that values at P0, D1, D2, D3, and D7 were significantly lower than Pre (*p* <.001, *η*² = 0.536), while D3 and D7 were higher than D1 and D2 (*p* <.001) (Fig. 2E).

For PPT_dist_, no interaction was detected (*p* =.501, *η*² = 0.031), but a significant main effect of group indicated lower values in ECC than EQI (*p* =.003, *η*² = 0.278). A main effect of time showed that PPT_dist_ decreased at all time points from P0 to D7 compared to Pre (*p* <.05, *η*² = 0.504) (Fig. 2F).

### Muscle damage markers

**Fig. 3:**
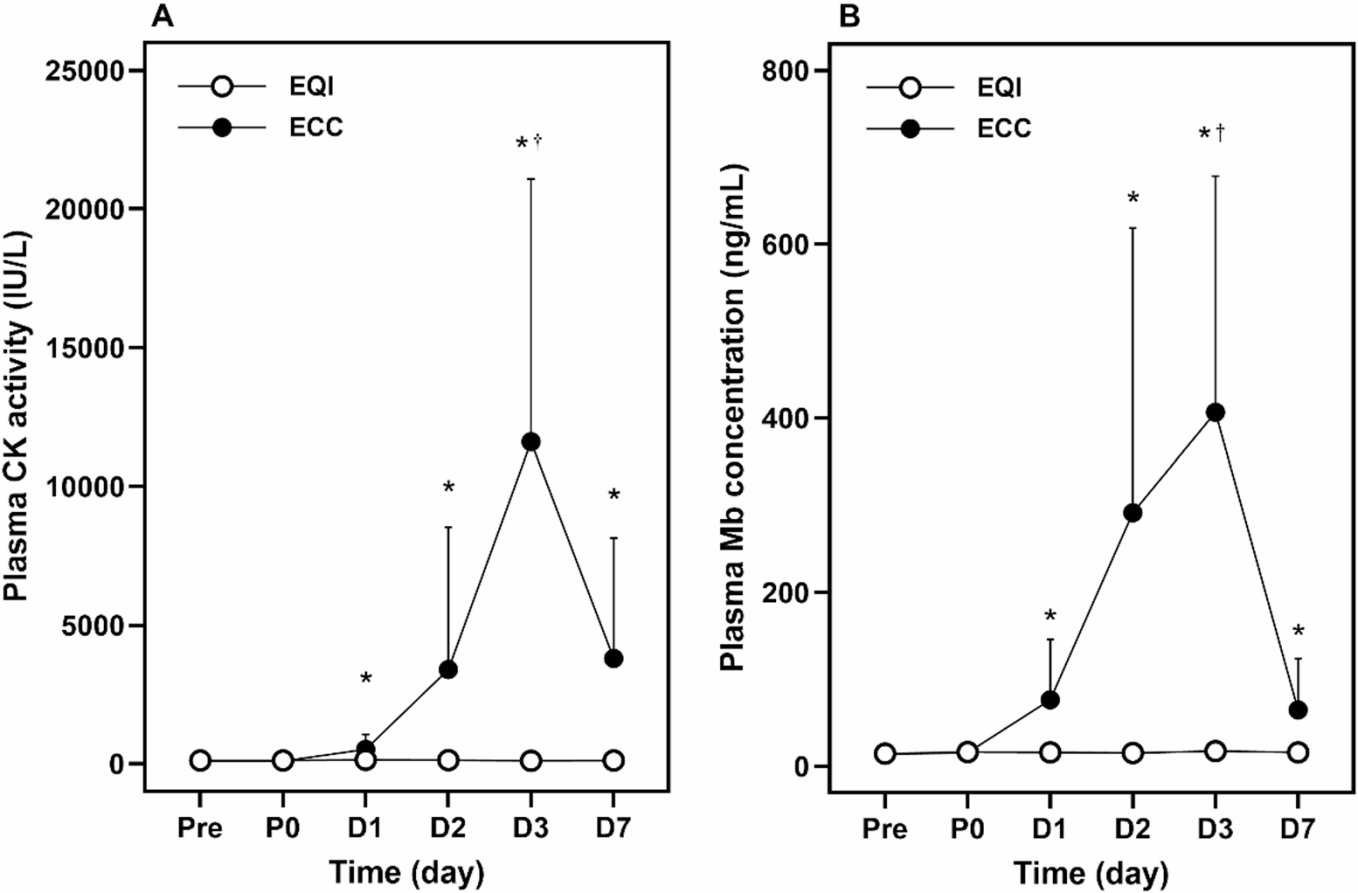
Changes (mean ± SD) in plasma creatine kinase (CK) activity (**A**) and myoglobin (Mb) concentration (**B**) from baseline (Pre) to immediately after (P0), and 1 (D1), 2 (D2), 3 (D3), and 7 (D7) days post-exercise in the eccentric (ECC) and eccentric quasi-isometric (EQI). ^*^ indicate significant differences between condition at the same time point (*p* <.05); ^†^ indicate significant differences compared to Pre within the same condition (*p* <.05).

A significant group× time interaction was observed for plasma CK activity (*p* <.001, *η*² = 0.368). CK levels were significantly higher in the ECC group than in EQI at D1 (*p* =.016, *d* = 1.201), D2 (*p* =.030, *d* = 0.886), D3 (*p* <.001, *d* = 1.681), and D7 (*p* =.005, *d* = 1.175). In ECC, CK at D3 (11,601 ± 9,483 IU/L) was significantly higher than Pre, D1, D2, and D7 (*p* <.05, *η*² = 0.546). No significant time effect was found in EQI (*p* =.122, *η*² = 0.132) (Fig. 3A).

A significant group× time interaction was also observed for plasma myoglobin concentration (*p* <.001, *η*² = 0.330). Mb levels were significantly higher in ECC than in EQI at D1 (*p* =.007, *d* = 1.201), D2 (*p* =.005, *d* = 1.173), D3 (*p* =.007, *d* = 1.986), and D7 (*p* =.009, *d* = 1.140). In ECC, Mb at D3 was significantly higher than Pre, P0, D1, and D7 (*p* <.001, *η*² = 0.507). No significant time effect was observed in EQI (*p* =.546, *η*² = 0.063) (Fig. 3B).

## Discussion

The present study showed that TUT during EQI was 3.1 to 4.4 times longer than during ECC. Despite the prolonged TUT, the EQI group exhibited smaller changes in muscle damage markers, including attenuated MVC loss, less ROM restriction, reduced CIR swelling, and lower serum CK and Mb levels. PPTdist was higher in EQI than in ECC, indicating less distal biceps discomfort. Most indicators returned to baseline by D7 in the EQI group.

EQI is characterized by alternating ISO holds and controlled ECC actions within each repetition, creating a continuous contraction sequence^12,14,22^. In the present study, total TUT during EQI was approximately 3.35 to 4.31 times longer than during ECC, consistent with previous findings in the knee extensors (EQI: 242 ± 132 s vs. ECC: 135 ± 72 s)^14^. This extended TUT reflects a higher cumulative mechanical stimulus, yet EQI did not result in substantial muscle damage. While the ECC group showed peak plasma CK (11,601 IU/L) and Mb (406 ng/mL) concentrations at D3, the EQI group exhibited no marked elevations in these markers. Despite the longer TUT, EQI induced smaller changes across all muscle damage indices compared to ECC, suggesting that the characteristics of the contraction, rather than its duration alone, influenced the physiological response. A potential explanation lies in the markedly slower ECC velocity observed during EQI. Prior studies report angular velocities around 1.29–1.34°·s⁻¹ for EQI^13,14^, substantially lower than conventional ECC loading. Faster ECC contractions are known to cause greater disruption to Z-lines, increased sarcolemmal permeability, and elevated CK and Mb levels, often contributing to delayed-onset muscle soreness^5,33,34^. Furthermore, even under matched torque-time integrals, ECC contractions have been shown to produce greater peripheral fatigue and muscle damage compared to ISO and CON modalities^3^. These findings support the hypothesis that the mode of tension application plays a more critical role in muscle damage than the total mechanical load applied^3,35^.

Differences in motor unit recruitment patterns between contraction types may also contribute to the extent of muscle damage. Due to the higher mechanical efficiency of ECC contractions, fewer motor units are recruited at a given load, thereby increasing the mechanical tension borne by each active fiber^6,36^. This disproportionately loads the initially recruited type II fibers, which are more susceptible to damage due to their weaker cytoskeletal support^37^ and shorter optimal length for force production^4^. As a result, ECC may expose fast-twitch fibers to excessive tension, leading to greater microstructural disruption. In contrast, EQI involves sustained force production to volitional fatigue, necessitating progressive recruitment of additional motor units to maintain force output^13^. This gradual activation pattern likely induces moderate neuromuscular fatigue while limiting instantaneous tension peaks. Indeed, previous work suggests that peak surface EMG during EQI may be lower than during isotonic ECC contractions^22^, indicating less acute neural drive. The more evenly distributed tension over time in EQI may help reduce abrupt strain on fast-twitch fibers. Taken together, the distinctive recruitment strategy of EQI allows for the accumulation of neuromuscular stimulus under lower per-fiber mechanical load, thereby attenuating structural damage^7^. This differential motor unit activation and tension distribution across the muscle length–tension curve may help explain the lower MVC loss and reduced damage marker responses observed following EQI.

In terms of ROM and CIR responses, the EQI group exhibited only a slight reduction in ROM at D1, with rapid recovery thereafter. This may reflect a lower degree of connective tissue disruption^1^. In contrast, the ECC group showed sustained ROM limitation through D7, potentially indicating more extensive connective tissue damage and increased passive stiffness. Reductions in ROM are often associated with microdamage to the fascia or tendon, which may alter tissue viscoelasticity and reduce extensibility^8^. Similarly, ECC-induced swelling was more prolonged, with CIR remaining elevated above baseline between D2 and D7, whereas the EQI group returned to baseline earlier, suggesting a lower inflammatory response or less fluid accumulation.

Regional differences in PPT were also observed. At the PPTprox and PPTmid regions of the biceps brachii, both groups showed comparable pain sensitivity. However, at the distal site (PPTdist), pain sensitivity was higher in the ECC group than in the EQI group. This finding is consistent with Oranchuk et al.^14^, who reported higher distal tenderness in the rectus femoris and vastus lateralis following ECC. The observed differences may relate to the regional distribution of mechanical tension during contractions. Recent mechanical analyses suggest that EQI primarily generates tension in the early phase of the ROM, targeting short to moderate muscle lengths^15^. In contrast, ECC has been associated with greater post-exercise tenderness in the distal and mid-muscle regions^30^. Thus, EQI appears to induce less structural disruption in the distal elbow flexors, resulting in attenuated pain in this region. Considering that pain can inhibit force production via neural mechanisms^8^, this may partly explain the prolonged strength recovery deficit observed in the ECC group. Notably, although PPTprox and PPTmid showed similar reductions in pain sensitivity across contraction modes, these responses may originate from different tissue-related sources. Reductions in PPT following ECC have been associated with microtrauma at the sarcomere or musculotendinous junction^4,6^, whereas EQI may reduce PPT through increased connective tissue tension or metabolite accumulation during sustained contractions^12,14^. Accordingly, while regional PPT responses partially overlapped between conditions, differences in tissue loading characteristics may contribute to distinct recovery profiles.

No previous studies have examined the acute muscle damage responses of the elbow flexors following EQI exercise, although one study has reported its effects on the knee extensors^14^. The present study is the first to show that EQI, characterized by ISO control and reduced ECC velocity, can attenuate muscle damage and inflammatory responses in the elbow flexors, potentially improving training safety and recovery. Despite a longer time under tension, the smaller changes in damage markers suggest that EQI provides a favorable balance between stimulus and mechanical stress. Notably, only the distal site exhibited lower pain sensitivity under EQI, suggesting a regional specificity in nociceptive responses. This observation implies that EQI may alter local mechanical loading or nociceptive sensitivity in a manner distinct from traditional ECC contractions. However, this study focused solely on acute responses. Its effects on metabolic stress, hypertrophy, and long-term adaptations remain unclear. Although Oranchuk et al.^12^ proposed that EQI may induce high metabolic stress, this has not been directly confirmed. A recent study by Henderson et al.^18^ compared EQI with isotonic training of the biceps brachii and found that both increased muscle size and strength, though the gains with EQI were smaller (hypertrophy: 4.0% vs. 6.7%; 1RM: 12.8% vs. 19.6%). These findings suggest that while EQI may not be optimal for maximizing hypertrophy, it appears to be a lower-damage strategy that still provides a meaningful neuromuscular stimulus.

In addition, this study contributes to the limited body of evidence regarding EQI training effects in the upper limbs. Previous EQI studies have primarily focused on the knee extensors, often using experimental designs matched for angular impulse^13–15^, while direct comparisons in upper-body muscles remain scarce. A preliminary study by Henderson et al.^22^ on the elbow flexors showed that EQI elicited prolonged time under tension and moderate neuromuscular fatigue. However, peak surface EMG activity was slightly lower compared to isotonic training, suggesting differences in neural activation patterns. Based on these findings, the present study confirms that EQI induces typical acute physiological responses in upper-body training without causing substantial muscle damage. This supports the hypothesis that EQI has low-damage potential. These results are consistent with, and expand upon, current understanding of ECC training mechanisms. Specifically, controlling ECC contraction characteristics, such as reducing contraction velocity or extending the duration of applied tension, can promote adaptation while minimizing structural disruption^3,35^. This supports a theoretical framework in which the manner of tension application, rather than its absolute magnitude, plays a key role in modulating muscle damage and adaptation. In this context, EQI represents an innovative eccentric contraction model that may help reduce the risk of EIMD while preserving the benefits of resistance training.

Although the present findings support that EQI can consistently induce prolonged time under tension and representative acute physiological responses in upper-limb training, without causing marked changes in muscle damage indicators such as ROM, CK, Mb, and pain, several limitations should be noted. The study simulated a practical dumbbell training context in which only the relative external load could be standardized. Accordingly, inferences are limited to volitional failure conditions. First, this study examined only acute responses following a single exercise session and does not allow inference about long-term adaptations. Second, the exercise involved single-joint elbow flexor movements, and thus the findings cannot be generalized to other muscle groups or multi-joint patterns. Third, dynamic assessment of tension distribution across joint angles was not conducted, limiting interpretation of how mechanical loading was distributed during EQI. Finally, no sex-based analysis was performed, which may have overlooked potential differences in TUT responses between males and females^22^. In terms of application, EQI is not suitable for contexts requiring high-speed or explosive movements. However, due to its stable force output and minimal muscle damage response, EQI may have potential use for untrained beginners during the early phases of resistance training^2,19,20^. Future research should investigate long-term adaptations, neuromuscular control mechanisms, and context-specific implementation strategies to establish a more comprehensive framework for the use of EQI.

## Conclusion

This study showed that EQI exercise induced longer TUT but resulted in smaller changes in muscle damage markers compared to ECC in the elbow flexors. EQI may serve as a low-damage alternative for upper-body resistance training, with region-specific reductions in pain sensitivity at the distal elbow flexors and markedly attenuated structural damage. Future studies are needed to explore long-term adaptations and practical implementation across different populations.

## Data Availability

The datasets used and/or analysed during the current study are available from the corresponding author on reasonable request.
